# Effect of Intravitreal Ranibizumab in the Treatment of Peripapillary Choroidal Neovascularisation

**DOI:** 10.1155/2011/602729

**Published:** 2011-12-22

**Authors:** Hassan Hamoudi, Torben Lykke Sørensen

**Affiliations:** Department of Ophthalmology, Copenhagen University Hospital Roskilde and University of Copenhagen, 4000 Roskilde, Denmark

## Abstract

Intravitreal ranibizumab therapy is widely used in treatment of subfoveal choroidal neovascularisation (CNV) in age-related macular degeneration. We wanted to study the effect of intravitreal ranibizumab therapy in peripapillary CNV. A prospective recording of treatment outcomes in twelve eyes (12 patients) with peripapillary CNV with intravitreal injections of ranibizumab was performed. The patients received a series of 3 injections 4–6 weeks apart, and then a new ophthalmic examination was made including OCT and further therapy was given if the peripapillary CNV was still active. Nine patients had idiopathic peripapillary CNV, and in 3 patients it was associated to age-related macular degeneration. Followup had to be at least 6 months. The mean follow-up time was 15.9 (range 9–27) months and the mean number of injections 6.2 (3–10). In 10 patients treatment had resulted in an inactivation of the peripapillary CNV, but 3 of them had reactivation, while 2 patients had no inactivation. Currently, 5 patients are continuous to receive treatment. VA improved in 10 patients. Intravitreal ranibizumab therapy appears to be effective in patients with peripapillary CNV, but in some cases there is repeated reactivation or continuous activity of the peripapillary CNV.

## 1. Introduction

The use of antivascular endothelial growth factor (anti-VEGF) agents for the treatment of retinal diseases is constantly increasing. Anti-VEGF was primarily introduced for the treatment of age-related macular degeneration (AMD) complicated with a sub- or juxtafoveal choroidal neovascularisation, but its use is now expanding to retinal vein occlusions and diabetic macular edema [1–3].

Peripapillary CNV is rare. Several conditions can cause peripapillary CNV including AMD (the most common cause, 45.2% of the cases), idiopathic (39.1%), multifocal choroiditis (4.3%), angioid streaks (2.6%), histoplasmosis (1.7%), choroidal osteoma (1.7%), optic disc drusen (0.9%), and congenital disc anomaly (0.9%) and has been reported in sarcoid [4]. No randomised or large study has evaluated the efficacy of anti-VEGF on peripapillary CNV hence standard care for peripapillary CNV remains argon laser photocoagulation or photodynamic therapy [5, 6]. However, since anti-VEGF is effective in treating CNV, it seems relevant to treat peripapillary CNV with anti-VEGF.

Information regarding the efficacy of anti-VEGF on peripapillary CNV is based on case reports and small case series [7–12]. Overall the results suggest a possible beneficial effect of anti-VEGF on peripapillary CNV, but questions remain regarding the long-term beneficial effect of the treatment, since continuous visual decline, and CNV activity despite treatment has been reported in some patients [7, 12].

The purpose of this study was to evaluate the efficacy of ranibizumab in patients with peripapillary CNV.

## 2. Methods

Twelve eyes (12 patients) were included in this study. At baseline all patients underwent an ophthalmic examination including a dilated fundus examination, best-corrected visual acuity (VA) using the early treatment of diabetic retinopathy study (ETDRS) chart, spectral domain optical coherence tomography (SD-OCT) imaging using Heidelberg HRA-Spectralis system (Heidelberg Engineering, Heidelberg, Germany) and fluorescein angiography (FA) as well as indocyanine green (ICG) angiography to rule out polypoidal vasculopathy was performed. The patients were initially assigned to 3 intravitreal injections of 0.5 mg ranibizumab 4–6 weeks apart.

Approximately, 1 month after the 3rd injection the patients returned for a clinical reevaluation including VA, SD-OCT, and dilated fundus examination. The OCT was both done through the fovea and the optic nerve. Based on signs of activity (intra- or subretinal fluid or retinal haemorrhages) it was decided whether to continue treatment using a variable dosing regimen—1 to 3 injections—or to reevaluate the patient again every 4–6 weeks. The reevaluation consisted of repeated VA, SD-OCT and dilated fundus examinations. We did not use FA to evaluate reactivation.

Informed consent was obtained from all participants and they all agreed to the off-label nature of the treatment. None of the patients had received any other treatment for peripapillary CNV.

## 3. Results

**Figure 1:**
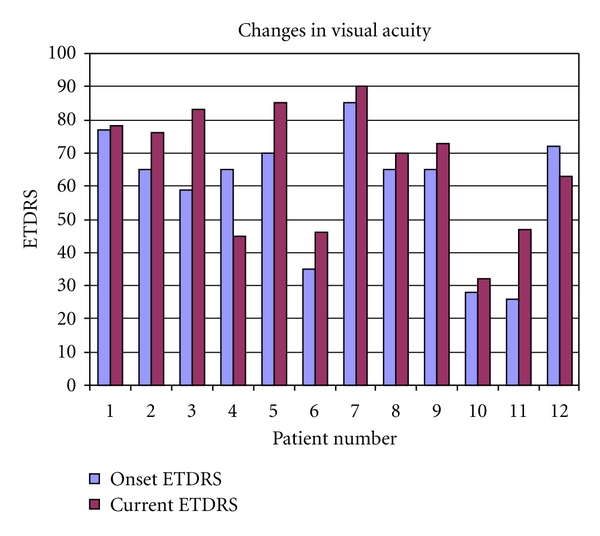
Changes in visual acuity after treatment.

**Figure 2:**
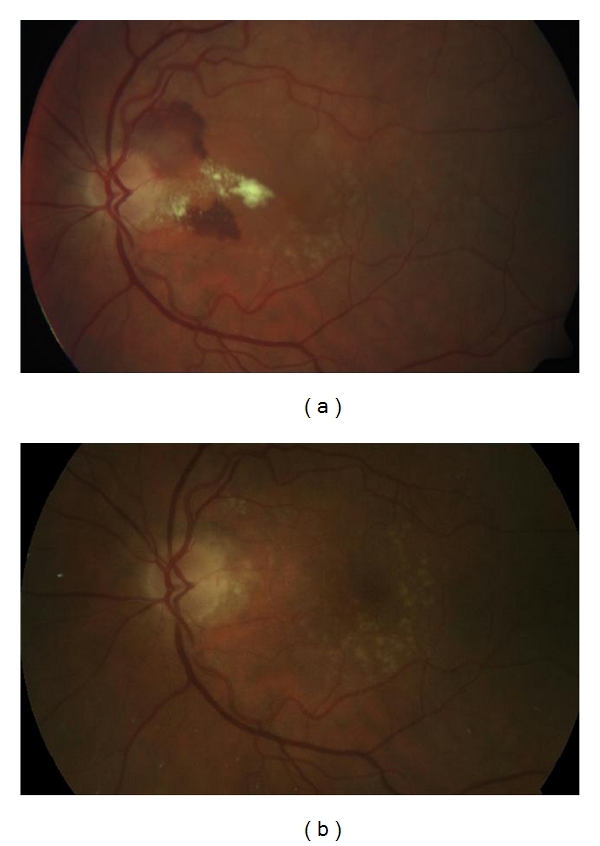
An example of fundus photography showing peripapillary CNV before and after treatment in a patient with AMD (patient 4).

Patient characteristics and treatment results are shown in Table 1 and Figure 1. All patients were Caucasian, with a mean age of 73 years (range: 28–87), 7 females and 5 males. The mean length of followup was 15.9 months (range: 9–27). The mean number of injections with intravitreal ranibizumab was 6.2 (range: 3–10). In patients without obvious signs of drusen and/or changes in the RPE the CNV was categorized as idiopathic. Nine eyes had idiopathic peripapillary CNV and 3 eyes had peripapillary CNV as part of AMD (Figure 2). All patients presented with subfoveal fluid causing a decrease of vision. The mean visual acuity at baseline was 59 letters (range: 26–85) and had significantly improved to 66 letters (range: 32–90) after treatment (*P* = 0.002, paired samples *t*-test). Ten out of 12 patients experienced an improvement in VA after treatment while vision declined in 2 patients.

Inactivation after the initial treatment regime with three injections was obtained in 5 patients, and of them 2 patients experienced reactivation after 8 and 16 months, respectively. In 5 other patients inactivation was obtained after 5–10 injections, of them 1 patient had reactivation after 18 months. Two patients had no inactivation and do receive continuous treatment. Currently, 7 patients show no sign of activity of the CNV, while 5 patients are still receiving treatment at the time of followup. In total 10 out of 12 patients experienced inactivation at some point of followup, and of them 3 patients had reactivation. The central retinal thickness (CRT) decreased in all but 1 patients (Table 1). All 3 patients with AMD had inactivation after 3, 5, and 6 injections, respectively. The patient with 3 injections had reactivation after 16 months. Of the 9 patients with idiopathic CNV, inactivation was achieved in 7 patients, of them 2 had reactivation, and 2 others did not obtain inactivation and do still receive treatment.

## 4. Discussion

Peripapillary CNV is rare and treatment studies of this condition consist primarily of short case reports and case series treating peripapillary CNV with either bevacizumab or ranibizumab.

Four reports on the effect of bevacizumab have been published [7–9, 11]. Two are small case series, 6 eyes and 4 eyes, respectively, and two are case reports adding up to 12 eyes in total with variable aetiologies to the peripapillary CNV [7–9, 11]. Follow-up ranges from 3 months to mean followup of 13 months. All studies report an initial favourable response to treatment, but in the study with the longest follow-up, inactivity was obtained in five of six eyes, and one eye did not respond to treatment [7]. These findings correspond to our findings where 10 out of 12 patients had inactivation. Even though the findings are similar, there are several differences in our study and the study of Figueroa and coworkers the patients were treated with bevacizumab, mean age was 67.8 years, and mean VA at baseline was 44 letters, and 2 patients had earlier been treated with surgical removal of the neovascular membrane [7]. There is also difference in the length of follow-up in their study compared to ours, since they had a mean followup of 13 months (range 6–16), and 2 of our patients had reactivation after 16 and 18 months of followup.

Only two papers have reported on the efficacy of ranibizumab in peripapillary CNV [10, 12]. One is a case series of seven eyes (6 patients) and one is a case report. The case series studied patients with paripapillary CNV of various aetiologies (4 angioid streaks, 2 idiopathic, 1 as part of AMD) with a mean followup of 12 months. Ranibizumab was successful in improving VA and resolution of subfoveal fluid was accomplished. [10] The case report studied one patient with sarcoidosis, uveitis, and peripapilliary CNV and after 12 months of followup there were still signs of activity [12].

Even though peripapillary CNV in some instances can be watched because of the asymptomatic nature, treatment should be considered when the fovea is threatened. Treatment of peripapillary CNV has recently been reviewed, and generally there is lack of evidence concerning the ideal treatment strategy in patients with peripapillary CNV, even though the theoretical rationale favours treatment with anti-VEGF [13].

In our study, we find a tendency towards efficacy of ranibizumab in the treatment of peripapillary CNV. However, complete inactivation of the CNV was only obtained in 7 of 12 patients (58%) at the end of followup. In the majority of patients, VA improves and the CRT decreases on OCT. Even though our study has a limited number of patients it is still the largest number of cases and the longest followup so far reporting on the efficacy of ranibizumab in peripapillary CNV. Our findings suggest that ranibizumab is a feasible solution for the treatment of peripaillary CNV, but reactivation and lack of inactivation resulting in a need for continuous treatment are common.

## Figures and Tables

**Table 1:** Patient characteristics and treatment results. (F: female; M: male). (Central retinal thickness).

Patient number	Sex	Age years	Diagnosis	Onset ETDRS	Current ETDRS	Number of injections	Onset CRT	Current CRT	Follow-up months	Current status
1	F	80	Idiopathic	77	78	10	302	235	22	Continuous treatment
2	M	74	Idiopathic	65	76	9	291	211	21	Continuous treatment
3	F	70	Idiopathic	59	83	3	461	210	14	Inactive
4	F	81	AMD	65	45	9	486	140	27	Continuous treatment
5	F	87	Idiopathic	70	85	3	274	245	18	Inactive
6	F	76	Idiopathic	35	46	3	400	292	23	Inactive
7	F	70	Idiopathic	85	90	9	280	280	17	Inactive
8	F	81	Idiopathic	65	70	6	615	543	9	Continuous treatment
9	M	76	AMD	65	73	6	348	280	15	Inactive
10	M	76	AMD	28	32	5	500	384	9	Inactive
11	M	28	Idiopathic	26	47	6	350	322	9	Inactive
12	M	81	Idiopathic	72	63	5	307	250	9	Continuous treatment
